# Obtaining Relief by False Pretences

**Published:** 1921-02-05

**Authors:** 


### Obtaining Relief by False Pretences.

Since our comments on the unfortunate results which have already flowed, and are continuing to flow, from the policy of giving indiscriminate relief in those areas in which local authorities have been subjected to pressure of a threatening kind by a certain type of unemployed we have received a good deal of information which goes to suggest that there is now a considerable tightening up of administration, and that borough councils and distress committees are acting more fearlessly, and therefore more fairly. Boards of Guardians are also endeavouring, it appears, to exercise more care in getting to the bottom of applications for relief, though several such bodies are finding that the work is becoming too heavy a task for the number of relieving officers at their disposal. It is due to the vigilance of these men, who are quite properly asking for assistance during the period of stress, that discovery is made of cases in which men are found applying for relief at more than one station, or in which others, as has been proved, have obtained relief both from borough councils and relief committees while still in work. When complaints of this kind are justified by the evidence local authorities ought not to hesitate to take proceedings in the courts against the offenders on the charge of obtaining relief by false statements. It is a particularly mean offence in the present crisis.

